# Basic essential education program (BEEP): a brief introductory faculty development course for medical teachers

**Published:** 2012-09-30

**Authors:** Robert Madan, Raed Hawa, Bruce Ballon, Ivan Silver, Stacey Bernstein

**Affiliations:** 1 Baycrest Centre for Mental Health, Toronto, Ontario, Canada; 2 Department of Psychiatry, University of Toronto, Toronto, Ontario, Canada; 3 Toronto Western Hospital, University of Toronto, Toronto, Ontario, Canada; 4 Departments of Child Psychiatry and Addiction Psychiatry, University of Toronto; 5 Centre for Addiction and Mental Health, University of Toronto; 6 Hospital for Sick Children, Toronto, Ontario, Canada; 7 Department of Paediatrics, University of Toronto

## Abstract

**Background:**

Physicians have a unique role in teaching future physicians and allied health professionals. Yet, most medical doctors have limited instruction in this critical component of their daily activity.

**Methods:**

This study was a prospective cohort study of the effectiveness of a local teaching program at two teaching hospitals for junior faculty. Based on a needs analysis and literature review, the teaching program was developed in an accessible and compact format of six consecutive, one-hour “lunch and learn” sessions, held locally over a six week period. Pre-post questionnaires and focus groups were used to evaluate the program.

**Results:**

Participants reported being satisfied with the course as whole, particularly in respect to the format and location. There was an improvement in their knowledge in all content areas covered. The greatest benefits were derived from fostering a community of practice and having the opportunity to role play and simulate teaching skills. An attitudinal change towards teaching was noted.

**Conclusions:**

A brief, local faculty development program was effective in enhancing physicians’ knowledge, skills, and attitudes in teaching.

## Introduction

Physicians in academic settings are increasingly expected to teach.^1^ Excellence in teaching is an important component of faculty promotion. Little instruction is provided to faculty in this important activity.^2^ New hires have unique teaching challenges and anxieties that are not addressed with the lack of available orientation programs.^3^–^4^

Faculty development programs at academic centres have been established to enhance the educational competency of clinicians. There is evidence that faculty development programs in education lead to participant satisfaction, perceived program usefulness and changes in attitudes, behaviours and knowledge.^5^–^7^

Barriers to participation in faculty development programs include a lack of awareness of the programs’ existence, enrolment restriction, lost income and lack of protected time.^8^–^9^ In an attempt to overcome these barriers, a local faculty development program was developed and implemented to improve the teaching competence and attitudes of junior medical staff at two academic health science centres. The following reports on the impact of a novel faculty development program that addresses these barriers.

## Methods

### Study design

This study was a prospective cohort study of the effectiveness of a local teaching program for junior faculty at two teaching hospitals. The program was called “Basic Essential Education Principles (BEEP): A Brief Faculty Development Course for Medical Teachers.” The objectives for the course included enhancing teaching skills and providing a forum for collegial exchange and reflection.

Based on a needs analysis, literature review, stakeholder interviews and departmental surveys, the teaching program was developed in an accessible and compact format of six consecutive, one-hour “lunch and learn” sessions, held locally over a six week period. The curriculum for the program is shown in Table 1. Our objective was to deliver a practical, accessible faculty development program.

### Setting

The program was held at two academic health sciences centres fully affiliated with the Faculty of Medicine at the University of Toronto with research ethics approval.

### Sample size

The target audience was junior medical faculty who were in the first 10 years of their career. There were seven participants at site A and five participants at site B for the first series. There were eleven participants at site A for the second series. There were not enough participants to run the program for the second series at site B.

### Sampling methods

Division chiefs/Program directors offered this opportunity to staff within their divisions. An e-mail was sent to all faculty who met the entry criteria, informing them about the program and recommending that interested parties contact their division head about enrolment.

### Study protocol

The program ran concurrently at both sites in October 2005 and at one site January 2006. The individual sessions were co-facilitated in pairs by the authors of this report.

The format was interactive and included mini-lectures, role play, case examples and reflective discussion. The participants were provided with pre-readings. Participants were asked to bring challenging scenarios to be discussed in the context of the session’s topic. Enduring materials were provided for easy reference. The program was accredited through the local university CME office for 6 hours of continuing education credits.

### Outcome measures

A mixed-methods case study approach was adopted to explore the impact of the BEEP program.^10^ Participants completed retrospective pre-post-test surveys at each session to measure impact.^11^ The ratings were based on a 5-point Likert scale, where 1 = unsatisfactory, 2 = needs improvement, 3 = good, 4 = very good, 5 = outstanding. The survey consisted of three questions: (1) I would rate my general knowledge of today’s topic as...; (2) I feel prepared to disseminate today’s concepts/ideas in my department; and (3) I see myself making use of the information that was provided in today’s class. Upon completion of the course, three focus groups involving the participants were conducted.

### Data analysis

Survey data were compiled and analyzed using SPSS version 15.0. Missing data were replaced with mean scores due to the small sample size. Means, standard deviations and frequencies were explored across each session and across each question. Pre-post comparisons were conducted using the Wilcoxon Signed Ranks test, a non-parametric test appropriate for ordinal data which is analogous to the paired sample t-test. Tests were adjusted for multiple comparisons using step-down Holm-Bonferonni corrections. Two-sided *p* ≤ 0.05 was regarded as statistically significant.

The focus group was audio-taped and the recordings transcribed and rendered anonymous by an independent research assistant. Both open and axial coding were employed to describe recurrent themes in the data.

## Results

**Figure 1:**
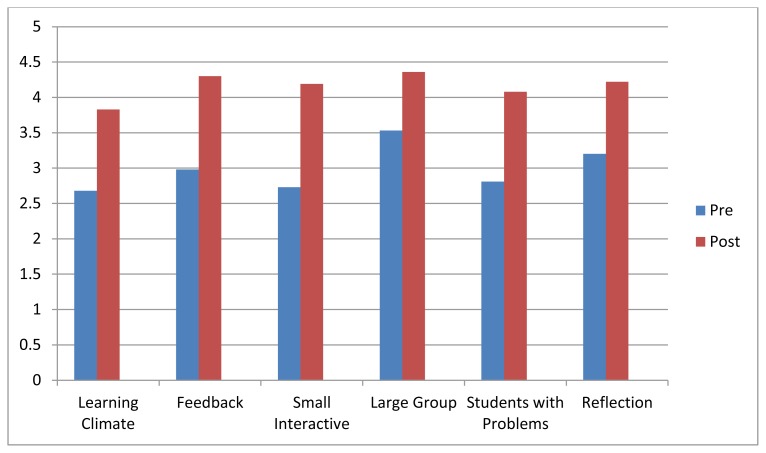
Mean pre-post scores per session

A total of 23 physicians participated in the course. There was variable attendance at site B. As such, for the quantitative analysis, data were collected ranging from 13 to 20 participants per session. For every session, respondents reported a significant improvement in mean pre-post scores (Figure 1). Respondents reported that they felt least knowledgeable about “The Learning Climate” before the intervention. They felt the most confident with “Teaching Large Groups” resulting in the least improvement for this topic. The greatest improvement was reported for “Small Interactive Teaching” (mean difference = 1.46).

The effect sizes from before and after the sessions ranged from 1.26 to 1.94. Further exploration of the mean scores within each session (general knowledge of the topic, feeling prepared to disseminate, envision making use of the material) revealed no significant difference between questions on the evaluation forms.

A total of three focus groups were conducted involving 14 participants. In total, all groups were very positive about their experiences in this program. People were interested in the program because of a self-perceived gap in knowledge and skills, a desire to improve, and because the program was designed for junior faculty. Participants stated that they were very satisfied with the gains made from the program. The topics were found to be relevant, logically sequenced, and combined a good balance of knowledge and skills. The groups reported learning a lot from the sharing of experiences. The dynamics of a small group were reported as beneficial. They appreciated the collegial environment and the provision of lunch at a convenient time and location. Finally, the cohesion of the participant groups was reported to be a strength. Sessions involving participant modeling of teaching skills were the most memorable and constructive. Endurable materials were greatly appreciated as the participants had not been exposed to these materials in the past.

It was reported that everyone appreciated the tangible knowledge, skills, and attitudes that they acquired. Changes in attitudes towards teaching were demonstrated by the following quotes:

- *The whole course was a source of inspiration- not to be afraid – have the courage to try new things.*
- *I learned patience – I am not afraid to challenge students.*
- *It gave me confidence regarding teaching – this has increased along with an increase in enthusiasm.*

## Discussion

According to the results for each core content area covered in the six sessions, the BEEP program was highly successful. The participants endorsed the structure, timing of the sessions, and brief duration. Many were inspired to learn more about teaching after having completed the program and felt empowered to apply new teaching methods. The opportunity to engage in simulations of teaching and having time to reflect was one of the most valued elements of the sessions.

Many teaching methods were used, so it is unclear which ones were the most effective in this format. Many of the participants reported that the use of simulations to practice techniques was helpful.

Program evaluations based on the retrospective pre-post surveys pose a limitation. It will be important with future iterations of this course to determine if the course had lasting effects in faculty’s teaching abilities. Future studies should determine whether a short faculty development program can improve teaching and student learning.

This study is limited as well by its small sample size and the difficulty in recruiting enough teachers from Academic Centre B. This centre was primarily a research-focused institution with less emphasis on teaching and education. Other challenges in Centre B included the fact that physicians were remunerated by fee for service and practice at two geographically separate and distinct sites, making attendance more difficult. Physicians in Centre A are salaried and all work within one large academic health science centre.

## Conclusion

This pilot study of six brief, one-hour faculty development sessions demonstrated that faculty appreciated the format and engaged with the teaching material. The program appeared to meet many of the faculty’s’ personal goals and was praised for its accessibility and efficiency. Participants wanted to learn more about teaching and suggested building a community of practice to allow participants to continue on with faculty development via peer supervision and support. It also appears that there may be institutional setting factors that need to be addressed to ensure recruitment and retention of faculty for these sessions.

The BEEP project appears to demonstrate that having a focused program aimed at maximizing engagement and address traditional barriers of attendance has merit in the spectrum of approaches and formats for faculty development.

## Figures and Tables

**Table 1:** BEEP curriculum

**Session 1: The Learning Climate**	General knowledge of the topic
	Prepared to disseminate concepts/ideas
	Envision making use of the information

**Session 2: How to Give Effective Feedback**	General knowledge of the topic
	Prepared to disseminate concepts/ideas
	Envision making use of the information

**Session 3: Small Interactive Teaching**	General knowledge of the topic
	Prepared to disseminate concepts/ideas
	Envision making use of the information

**Session 4: Teaching Large Groups**	General knowledge of the topic
	Prepared to disseminate concepts/ideas
	Envision making use of the information

**Session 5: Students with Problems**	General knowledge of the topic
	Prepared to disseminate concepts/ideas
	Envision making use of the information

**Session 6: Reflection**	General knowledge of the topic
	Prepared to disseminate concepts/ideas
	Envision making use of the information
